# A lactate‐responsive gene signature predicts the prognosis and immunotherapeutic response of patients with triple‐negative breast cancer

**DOI:** 10.1002/cai2.124

**Published:** 2024-05-17

**Authors:** Kaixiang Feng, Youcheng Shao, Jun Li, Xiaoqing Guan, Qin Liu, Meishun Hu, Mengfei Chu, Hui Li, Fangfang Chen, Zongbi Yi, Jingwei Zhang

**Affiliations:** ^1^ Department of Breast and Thyroid Surgery, Zhongnan Hospital of Wuhan University, Hubei Key Laboratory of Tumor Biological Behaviors Hubei Cancer Clinical Study Center Wuhan China; ^2^ Department of Pathology and Pathophysiology, Hubei Provincial Key Laboratory of Developmentally Originated Disease, TaiKang Medical School (School of Basic Medical Sciences) Wuhan University Wuhan China; ^3^ Department of Human Anatomy, TaiKang Medical School (School of Basic Medical Sciences) Wuhan University Wuhan China; ^4^ Department of Radiation and Medical Oncology, Zhongnan Hospital of Wuhan University, Hubei Key Laboratory of Tumor Biological Behaviors Hubei Cancer Clinical Study Center Wuhan China

**Keywords:** immunotherapy, lactate, risk model, subtype, TNBC

## Abstract

**Background:**

Increased glycolytic activity and lactate production are characteristic features of triple‐negative breast cancer (TNBC). The aim of this study was to determine whether a subset of lactate‐responsive genes (LRGs) could be used to classify TNBC subtypes and predict patient outcomes.

**Methods:**

Lactate levels were initially measured in different breast cancer (BC) cell types. Subsequently, MDA‐MB‐231 cells treated with 2‐Deoxy‐d‐glucose or l‐lactate were subjected to RNA sequencing (RNA‐seq). The gene set variation analysis algorithm was utilized to calculate the lactate‐responsive score, conduct a differential analysis, and establish an association with the extent of immune infiltration. Consensus clustering was then employed to classify TNBC patients. Tumor immune dysfunction and exclusion, cibersort, single‐sample gene set enrichment analysis, and EPIC, were used to compare the tumor‐infiltrating immune cells between TNBC subtypes and predict the response to immunotherapy. Furthermore, a prognostic model was developed by combining 98 machine learning algorithms, to assess the predictive significance of the LRG signature. The predictive value of immune infiltration and the immunotherapy response was also assessed. Finally, the association between lactate and various anticancer drugs was examined based on expression profile similarity principles.

**Results:**

We found that the lactate levels of TNBC cells were significantly higher than those of other BC cell lines. Through RNA‐seq, we identified 14 differentially expressed LRGs in TNBC cells under varying lactate levels. Notably, this LRG signature was associated with interleukin‐17 signaling pathway dysregulation, suggesting a link between lactate metabolism and immune impairment. Furthermore, the LRG signature was used to categorize TNBC into two distinct subtypes, whereby Subtype A was characterized by immunosuppression, whereas Subtype B was characterized by immune activation.

**Conclusion:**

We identified an LRG signature in TNBC, which could be used to predict the prognosis of patients with TNBC and gauge their response to immunotherapy. Our findings may help guide the precision treatment of patients with TNBC.

## Graphical abstract

The study was primarily conducted in three sequential steps: Step 1 involved generating a gene signature consisting of 14 lactate‐responsive genes, Step 2 focused on exploring the features associated with this lactate‐responsive gene signature, and Step 3 aimed to construct a consensus subtyping and risk evaluation system based on this gene signature.

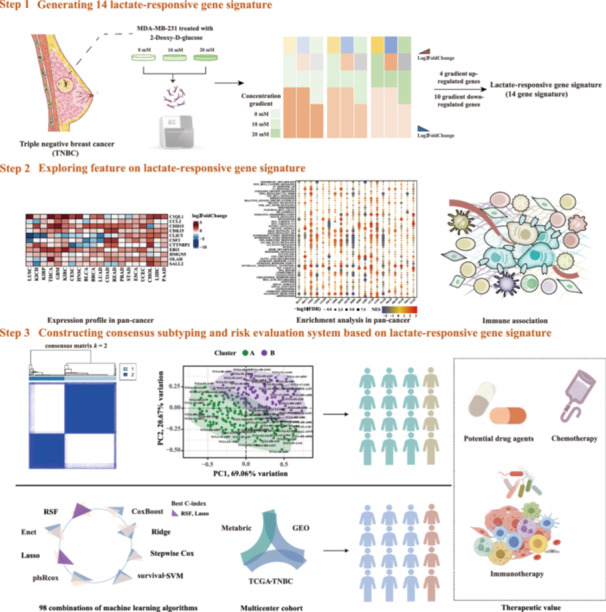

**Abbreviations**
2‐DG: 2‐deoxy‐d‐glucose
BC: breast cancer
DEG: differentially expressed gene
GEO: Gene Expression Omnibus
GO: Gene Ontology
GSEA: gene set enrichment analysis
GSVA: gene set variation analysis
IL‐17: interleukin‐17
KEGG: Kyoto Encyclopedia of Genes and Genomes
LRG: lactate‐responsive gene
MDSC: myeloid‐derived suppressor cell
METABRIC: Molecular Taxonomy of Breast Cancer International Consortium
MsigDB: Molecular Signatures Database
OS: overall survival
RNA‐seq: RNA sequencing
RSF: random survival forest
ssGSEA: single‐sample GSEA
TCGA: The Cancer Genome Atlas
TIDE: tumor immune dysfunction and exclusion
TME: tumor microenvironment
TNBC: triple‐negative breast cancer
WGCNA: weighted gene co‐expression network analysis

## 1 INTRODUCTION

Breast cancer (BC) remains a serious threat to human health. Nearly 310,000 new cases of BC and 43,000 BC‐associated fatalities were reported in the United States in 2024 alone [1]. The diverse characteristics of BC tumors and the lack of viable treatment options contribute to the development of triple‐negative BC (TNBC), the most lethal and frequently recurring BC subtype [2]. Various anticancer strategies have been developed to treat BC, including chemotherapy, targeted therapy, and immunotherapy. However, surgical resection and cytotoxic chemotherapy are still the most effective treatment options for TNBC [3]. Owing to the lack of clear symptoms, many patients with TNBC are diagnosed late and have high rates of recurrence and metastasis; this contributes to poor prognosis and a shortened survival time [4]. An increased understanding of TNBC biology and the advancement of multi‐omics approaches and other technologies offers increased possibilities for expanding the diagnostic and treatment options available to patients. In addition, better classification of TNBC subtypes and characterization of the diverse biological drivers will help the implementation of personalized treatment for TNBC [3, 4, 5].

TNBC has a distinct metabolic phenotype, which is characterized by more pronounced cellular metabolic dysregulation than other BC subtypes. For instance, TNBC cells have a preference for glycolysis, which is strongly associated with a worse prognosis [6, 7]. In accordance, pyruvate and lactate levels are significantly elevated in TNBC versus hormone receptor‐positive BC [8, 9]. Lactate, the end product of glycolysis, has traditionally been regarded solely as a metabolic byproduct. However, since the discovery of the Warburg effect in 1956, extensive research has revealed that lactate also serves as a signaling molecule in crucial physiological processes within cancer cells. For instance, it uses several mechanisms, including cell cycle regulation, immunosuppression, and energy metabolism, to promote cancer progression [10, 11]. The profound impact of lactate on the tumor microenvironment (TME) has prompted investigations into its role in tumor immunity. For instance, studies have demonstrated that lactate promotes BC growth by inhibiting dendritic cells from presenting tumor‐specific antigens to other immune cells [12]. In addition, it mediates interactions between tumors and macrophages to facilitate metastasis in BC [13] and exerts immunosuppressive effects on various type of immune cells, including natural killer cells and CD4^+^/CD8^+^ T cells [14, 15]. Moreover, lactate can regulate immune cell function within tumors by promoting the posttranslational modification of proteins through lactylation [16, 17, 18]. Therefore, exploring genes related to lactate metabolism may help the development of immunotherapeutic strategies against TNBC. Although immunotherapy drugs such as immune checkpoint inhibitors are available for the treatment of TNBC, their efficacy is limited by tumor heterogeneity (e.g., variation in the metabolic phenotypes among tumor cells within the TME and different patterns of immune infiltration) [2]. Therefore, the classification on tumors based on the expression of lactate**‐**responsive genes (LRGs) may help the selection of patients with TNBC for immunotherapy.

In our study, we used RNA sequencing (RNA‐seq) to identify 14 LRGs in MDA‐MB‐231 cells treated with 2‐deoxy‐d‐glucose (2‐DG) or l‐lactate. We then used this LRG signature to classify TNBC into two subtypes. Next, we generated and tested a model to predict the prognosis of patients with TNBC based on their lactate metabolism. Additionally, we characterized infiltrating immune cells in each TNBC subtype and determined how this parameter was associated with the response to immunotherapy. Our research showed the role of lactate metabolism in TNBC and demonstrated how it could be used to predict the prognosis and treatment response of patients with this aggressive form of BC.

## 2 MATERIALS AND METHODS

### 2.1 Data acquisition and preprocessing

Human BC RNA‐seq data were acquired from The Cancer Genome Atlas (TCGA) using the TCGAbiolinks R package [19]. The clinical survival data were obtained from TCGA pan‐cancer atlas. Additional expression profile data were obtained from the Molecular Taxonomy of Breast Cancer International Consortium (METABRIC) [20] and Gene Expression Omnibus (GEO) database [21]. Our study excluded patients who had a survival time of less than 30 days or missing data.

### 2.2 Selection of lactate‐metabolism‐related gene sets

To collect gene sets related to lactate metabolism, molecular signature database (MsigDB) [22] was interrogated using the keywords “lactate” and “lactic acid.” After merging and eliminating duplicate genes, 325 genes belonging eight gene sets related to lactate metabolism were obtained (Supporting Information: Table S1).

### 2.3 Gene Ontology (GO) and Kyoto Encyclopedia of Genes and Genomes (KEGG) analyses of RNA‐seq data

The differentially expressed genes (DEGs) with a significance level of *p* < 0.05 and log_2_ |fold‐change| > 1 were identified using the DESeq2 R package. GO and KEGG pathway enrichment analyses of the DEGs were performed using the “Clusterprofiler [23]” R packages and Metascape [24].

### 2.4 Gene set analysis using gene set enrichment analysis (GSEA) and single‐sample GSEA (ssGSEA)

The “Clusterprofiler” R packages were used to conduct GSEA. The cancer hallmark gene set was downloaded from MSigDB. Unlike GSEA, ssGSEA does not rely on grouping but calculates the score for each sample. Hence, the proportion of infiltrating immune cells and the LRG scores were calculated using the ssGSEA method implemented in the “gene set variation analysis (GSVA)” R package [25].

### 2.5 Cell lines and culture

The MCF‐10A cell line, which is derived from breast endothelial cells, along with the MCF‐7 and the 4T1 cell lines, were acquired from the Cell Bank of Shanghai Institutes of Biological Sciences in Shanghai, China. Additionally, the MDA‐MB‐231 and MDA‐MB‐468 cell lines were obtained from Wuhan Procell Life Technology Co. Ltd. MCF‐10A was grown in a dedicated unique medium (Procell). Additional BC cell lines were grown in DMEM (Invitrogen) supplemented with 10% fetal bovine serum (GIBCO) and 1% penicillin/streptomycin (GIBCO). The cells were cultured in at 37°C, 5% CO_2_.

### 2.6 Lactate assay

The lactate concentration in the cell supernatant was measured using the l‐lactate colorimetric assay kit (Elabscience), according to the manufacturer's instructions. In short, cells were cultured in six‐well dishes for 24 h. Next, the medium was substituted with fresh medium, and the cells were incubated for an additional 24 h. Finally, the supernatants were collected and their lactate levels were analyzed at 530 nm using a UV‐vis spectrophotometer (Thermo Fisher Scientific). The lactate concentration per cell was calculated by counting the number of cells assayed.

### 2.7 Cell Counting Kit‐8 (CCK‐8) assay

MDA‐MB‐231 cells seeded into 96‐well plates at ~1 × 10^3^ cells/well, cultured overnight, and then exposed to different treatment conditions. The evaluation was performed every 24 h and the medium in wells awaiting detection was replaced with medium containing 10% CCK‐8 reagent. The cells were cultured at 37°C for a further 2 h, before absorbance was measured at 450 nm.

### 2.8 RNA‐seq

MDA‐MB‐231 cells were exposure to 2‐DG (GlpBio) or sodium l‐lactate (Sigma‐Aldrich) and then subjected to RNA‐seq analysis. Briefly, MDA‐MB‐231 cells were treated with 2‐DG (10 or 20 mmol/L), l‐lactate (10 or 20 mmol/L), or phosphate‐buffered saline (control) for 24 h. Total RNA was extracted from three sample replicates for each condition using an RNA mini kit (Qiagen). The RNA quality was assessed using a combination of gel electrophoresis and Qubit (Thermo). RNA‐seq was performed on an Illumina Novaseq. 6000 instrument by DIATRE Biotechnology.

### 2.9 Drug discovery based on gene expression profile similarity

Drug prioritization analysis of lactate‐responsive DEGs was conducted using DREIMT [26], a drug discovery tool that detects similarity between expression profiles and drug prioritization feature, which prioritized using *τ* and drug specificity score and filtered based on statistical significance (false discovery rate < 0.05), was developed to explore the most suitable drugs for enhancing or suppressing the expression of user‐defined LRGs of interest.

### 2.10 Clustering of TNBC subtypes based on LRGs

An R implementation of the ConsensusClusterPlus [27] was utilized to perform consensus clustering based on LRGs. Specifically, the WardD2 algorithm with Euclidean distances (*k* = 2:10) was employed for conducting the consensus clustering analysis.

### 2.11 Evaluation of immunotherapeutic efficacy

Immunotherapy is a promising treatment for TNBC. Tumor immune dysfunction and exclusion (TIDE) [28] and clinical data sets (IMvigor210 cohort and GSE78220) were used to evaluate the role of LRGs. Next, the immune dysfunction and exclusion scores were generated for the activated and suppressed immune cells. Given the lack of data sets from TNBC patients receiving immunotherapy, a data set from patients with advanced urothelial cancer (IMvigor210 cohort) [29], who were treated with the anti‐PD‐L1 antibody (atezolizumab) (GSE78220) [30], was also included in this study.

### 2.12 Immune cell infiltration analysis

To ensure the consistency of the aforementioned results using various immune assessment algorithms, multiple immune cell gene sets (TIMER [31], MCP‐counter [32], Cibersort [33], and EPIC [34]) were employed and compared using ssGSEA and 28 immune cell gene sets, with the assistance of the R package “IOBR” [35].

### 2.13 Weighted gene co‐expression network analysis (WGCNA) to identify immune subtypes and co‐expression gene modules

The “WGCNA” [36] R package was used to identify the gene modules associated with certain immune subtypes. A total of 8000 genes were filtered based on a mean absolute deviation value of >0.1. The “pickSoftThreshold” function was then used to determine the ideal soft threshold power beta. A co‐expression network was generated by using the one‐step network approach. The co‐expression modules were then detected using the “blockwiseModules” function. The “plotDendroAndColors” and “labeledHeatmap” functions were used to construct a dendrogram and determine module–trait associations.

### 2.14 Prognostic model construction

Previously published machine learning algorithms, including random survival forest (RSF), Lasso, Enet, survival‐SVM, stepwise Cox, CoxBoost, plsRcox, and Ridge, were combined to create a prognostic model exhibiting excellent stability and accuracy [37]. The C‐index was calculated for all the validation data sets.

### 2.15 Survival analysis

The Kaplan–Meier method was used to examine the predictive significance of the risk score associated with immune‐related genes for overall survival (OS). The patients were categorized into high‐risk and low‐risk groups based on the median risk score value. Subsequently, the OS difference between the two groups of TNBC patients was calculated and visualized using the “Survival” and “Survminer” R packages.

### 2.16 Statistical analysis

Statistical analysis was performed using R software (version 4.2). A *p* < 0.05 was defined as a measure of statistical significance. The log‐rank test and Wilcoxon rank‐sum test were utilized to generate the Kaplan–Meier curves and assess differences between two groups, respectively. The *χ* ^2^ test was utilized to examine the differences between two categorical variables.

## 3 RESULTS

### 3.1 High rate of lactate production is a hallmark of TNBC

**Figure 1:**
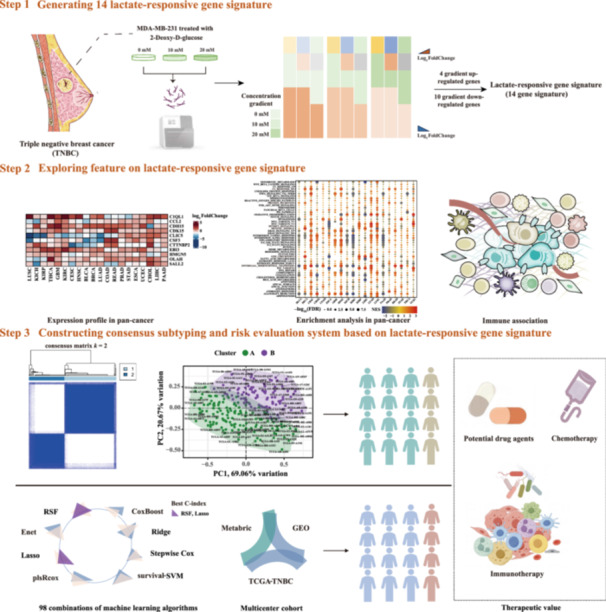
Schematic illustration of the study workflow. GEO, Gene Expression Omnibus; RSF, random survival forest; TCGA, The Cancer Genome Atlas; TNBC, triple negative breast cancer.

This study consisted of three main parts as follows: (1) Generating the 14‐LRG signature; (2) Characterizing the 14‐LRG signature; and (3) Constructing a consensus subtyping and risk evaluation system based on the 14‐LRG signature (Figure 1).

**Figure 2:**
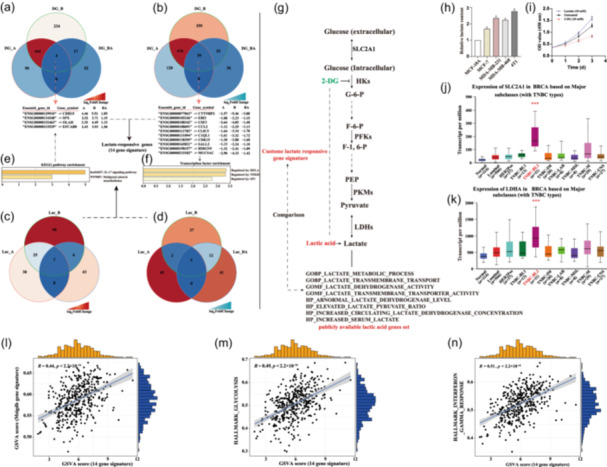
High rate of lactate production is a hallmark of triple‐negative breast cancer (TNBC). Venn diagram of the common upregulated (a) and downregulated (b) differentially expressed genes (DEGs) in MDA‐MB‐231 cells exposed to different concentrations of 2‐deoxy‐d‐glucose (2‐DG). Venn plot of the common upregulated (c) and downregulated (d) DEGs in MDA‐MB‐231 cells exposed to different concentrations of l‐lactate. (e, f) Gene Ontology (GO) and Kyoto Encyclopedia of Genes and Genomes (KEGG) enrichment and transcription factor analyses of the DEGs. (g) Schematic representation of the lactate synthesis pathway. (h) Levels of lactate in different BC cell lines. (i) Cell Counting Kit‐8 (CCK‐8) assessment of MDA‐MB‐231 cell growth following exposure to 2‐DG or l‐lactate. (j, k) Expression levels of glucose transporter and lactate dehydrogenase in different subtypes of BC. (l–n) Correlations between the 14 lactate‐responsive genes (LRGs) and the Molecular Signatures Database (MsigDB) lactate and cancer hallmark gene sets.

We began by exploring the lactate level based on the three key enzyme nodes in the glycolysis process (Figure 2g). We found that lactate levels gradually increased in the MCF‐7, MDA‐MB‐231, MDA‐MB‐468, and 4T1 cell lines relative to those of the non‐BC cell line MCF‐10A (Figure 2h). Moreover, the change in lactate level affected the proliferation of MDA‐MB‐231 cells (Figure 2i). These findings suggested that the high level of lactate was a prominent feature of TNBC cell lines and a factor promoting TNBC progression. We next used TCGA BC data set to determine the expression of SLC2A1 and LDHA, which is indirectly associated with changes in lactate levels. The above results confirmed that genes associated with lactate metabolism (e.g., *SLC2A1* and *LDHA*) were significantly upregulated in TNBC versus other BC subtypes (Figure 2j,k).

We next performed RNA‐seq of the 2‐DG‐ and lactate‐treated MDA‐MB‐231 cells, and showed that 14 genes (i.e., *CDH15*, *SPX*, *OLAH*, *EFCAB8*, *CTTNBP2*, *EBI3*, *CSF3*, *CCL2*, *CLIC5*, *C1QL1*, *CDK15*, *SALL2*, *HMGN5*, and *MUC5A*C) were differentially expressed between the two conditions (Figure 2a–d). The enrichment analysis revealed that the DEGs were mainly enriched in the interleukin‐17 (IL‐17) signaling pathway and malignant pleural mesothelioma. These genes were controlled by the transcription factors RELA, NFKB1, and SP1 (Figure 2e,f). To demonstrate the robustness of the gene set, we performed a correlation analysis between the 14‐LRG signature and the MsigDB lactate (Figure 2l) and cancer hallmark (Figure 2m,n) gene sets. We found that the LRGs identified primarily participated in immune regulation. This result prompted us to next investigate how lactate levels modulated the immune response.

### 3.2 The expression of most LRGs was dysregulated in TNBC and significantly associated with pan‐cancer

**Figure 3:**
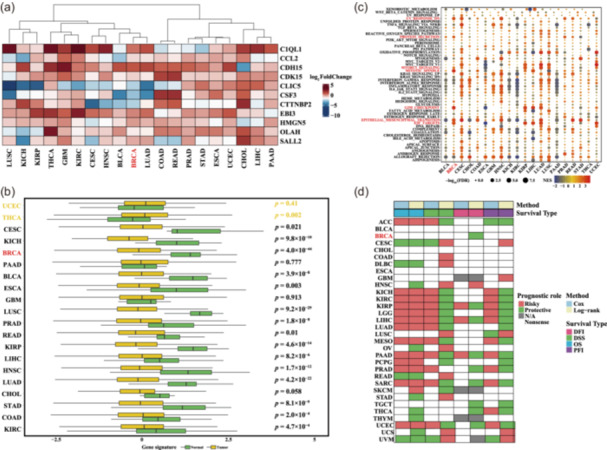
Expression of most lactate‐responsive genes (LRGs) was dysregulated and significantly associated with pan‐cancer. (a) Differential expression of LRGs in pan‐cancer and adjacent tissues. (b) The LRG lactate scores in pan‐cancer and adjacent tissues. (c) Enrichment of lactate score in 50 cancer hallmark gene sets. (d) Association between lactate score and pan‐cancer prognosis.

We next determined how the expression of the 14 LRGs differed between pan‐cancer and para‐cancer tissues. We found that *C1QL1*, *CCL2*, *CDH15*, *CDK15*, *EBI3*, *HMGN5*, *OLAH*, and *SALL2* were upregulated in TCGA BC, whereas *CLIC5*, *CSF3*, and *CTTNBP2* were downregulated (Figure 3a). We utilized the GSVA to calculate the gene set scores, before examining the differential expression of the genes of interested between pan‐cancer and the corresponding adjacent tissues.

We found that the lactate scores of the neighboring tissues were considerably higher than those of the cancerous tissues (Figure 3b). Next, we calculated the median lactate score and obtained the MSigDB cancer hallmark data set for GSEA analysis. We found that the UV_RESPONSE_DN genes were significantly upregulated in BC (Figure 3c). Notably, an inverse relationship was observed between lactate score and the prognostic risk index (Figure 3d). Further analysis revealed that the majority of the LRGs exhibited abnormal expression patterns and were linked to malignant biological activities such as excessive cellular growth and the epithelial‐mesenchymal transition. However, unexpectedly, the LRG signature was associated with a better prognosis. The mechanism underlying these findings requires further elucidation.

### 3.3 LRGs categorized TNBC into two distinct subtypes

**Figure 4:**
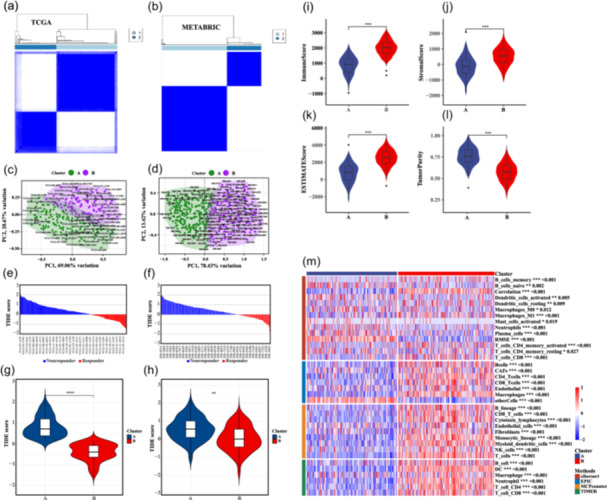
Lactate‐responsive genes (LRGs) categorized triple‐negative breast cancer (TNBC) into two subtypes. TNBC subtype analysis was based on the expression of LRGs in two data sets: The Cancer Genome Atlas (TCGA) (a) and Molecular Taxonomy of Breast Cancer International Consortium (METABRIC) (b). Principal component analysis was used to verify the accuracy TCGA (c) and METABRIC (d) data set typing. (e–h) The tumor immune dysfunction and exclusion (TIDE) algorithm was used on TCGA and METABRIC data sets to explore the responsiveness of TNBC subtypes to immunotherapy. (i–l) Tumor immune purity scores of the two TNBC subtypes. (m) cibersort, TIMER, MCPcounter, and EPIC were used to identify differences in the infiltrating immune cells between the two TNBC subtypes. *p < 0.05, **p < 0.01, ***p < 0.001, ****p < 0.0001.﻿﻿﻿﻿

We next demonstrated the classification value of LRGs. Using data from TCGA and METABRIC data sets, we showed that the LRGs segregated the TNBC into two subtypes (A and B) (Figure 4a,b). Principal component analysis was then used to verify the subtyping accuracy (Figure 4c,d).

Next, we investigated how the expression of LRGs was associated with variations in immune response. To this end, the immune cell score and the amount of immune cell infiltration among immune subtypes were calculated by ssGSEA. We obtained a total of 28 immune gene sets. The immune infiltration score differed significantly between the quiescent and mixed immune subtypes, with the latter subtype being more enriched in activated immune cells (Figure 4d). TIDE was the used to evaluate the responsiveness of the TNBC subtypes to immunotherapy. The data revealed that Subtype B had a lower TIDE score than Subtype A, indicating that immunotherapy may be more effective against Subtype B (Figure 4e–h). A greater ImmuneScore or StromalScore indicates a higher percentage of immune or stromal elements in the TME, respectively. In addition, we noticed that Subtype B had a significantly higher ImmuneScore, StromalScore, and ESTIMATEScore, and lower TumorPurity (Figure 4i–l). Next, we employed various techniques, such as cibersort, EPIC, MCPcounter, and TIMER, to characterize the tumor‐infiltrating immune cells. As anticipated, TNBC Subtype B was associated with higher numbers of infiltrating anticancer immune cells (including CD8^+^ T cells, activated CD4^+^ memory T cells, and memory B cells) than Subtype A (Figure 4m).

**Figure 5:**
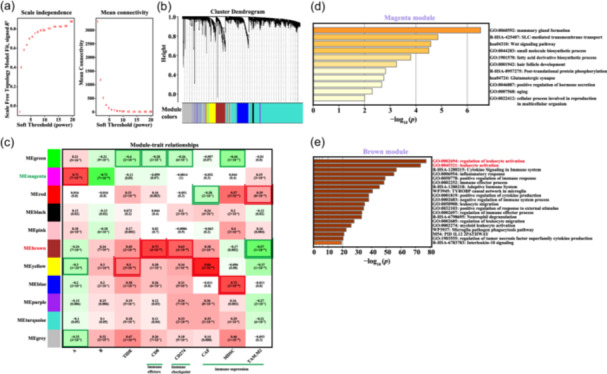
Correlation and enrichment analyses of the two triple‐negative breast cancer (TNBC) subtypes using the weighted gene co‐expression network analysis (WGCNA) method. (a) Determination of soft‐thresholding power in the WGCNA. (b) WGCNA dendrogram and gene modules selected. (c) Heatmap showing the correlation of WGCNA module with TNBC subtype. (d, e) Kyoto Encyclopedia of Genes and Genomes (KEGG) pathway and Gene Ontology (GO) enrichment analyses of WGCNA modules.

We next utilized the WGCNA to identify modules enriched in the TNBC subtypes; the sample dendrogram and soft threshold are shown in Figure 5a–c. We determined that the co‐expression network contained 12 modules. The Magenta and Brown modules were significantly negatively and positively correlated with the B module, respectively (Figure 5c). Mammary gland formation (GO:0060592), SLC‐mediated transmembrane transport (R‐HSA‐425407), and the Wnt signaling pathway (hsa04310) were negatively correlated with the B module (Figure 5d), whereas regulation of leukocyte activation (GO:0002694) and leukocyte activation (GO:0045321) were positively correlated with the B module (Figure 5e).

### 3.4 LRGs are good risk predictors for TNBC

**Figure 6:**
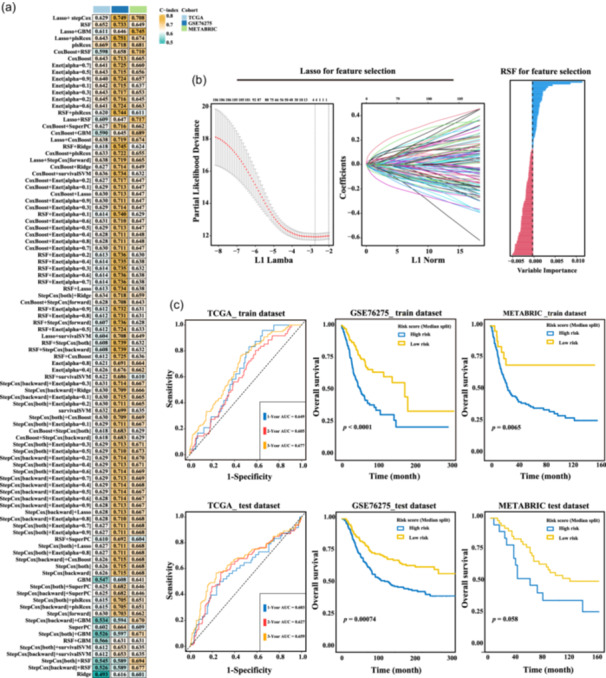
Lactate‐responsive genes (LRGs) serve as reliable predictors for the risk of triple‐negative breast cancer (TNBC). (a) A total of 98 prediction models were used in the LOOCV framework; each model's C‐index was then computed across all validation data sets. (b) In The Cancer Genome Atlas (TCGA)‐TNBC cohort, the optimal *λ* was determined by finding the minimum value of the partial likelihood deviance, which was then used to generate Lasso coefficients and random survival forest (RSF) for the most valuable prognostic genes. (c) Kaplan–Meier plots displaying OS in the TCGA‐TNBC, Molecular Taxonomy of Breast Cancer International Consortium (METABRIC), and GSE76275 data sets, calculated using the risk model.

An integrative machine learning procedure was next used to create a risk prediction system for immune suppression and activation based on the expression profiles of the 14 LRGs. We fitted eight types of prediction model to the TCGA‐TNBC training data set, before calculating the C‐index across all validation data sets. Notably, RSF and Lasso emerged as the most effective models, with the highest average C‐indexes (Figure 6a). The minimum value of the partial likelihood deviance was reached (Figure 6b), indicating the optimal *λ* in the Lasso regression.

**Figure 7:**
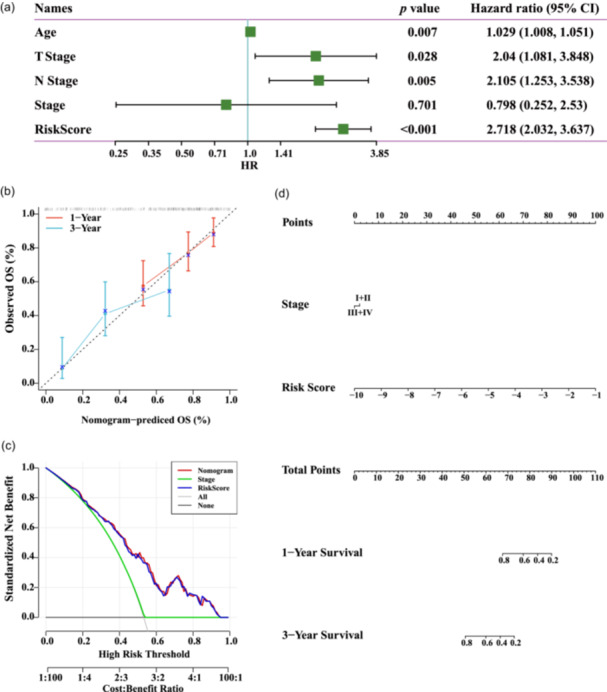
Risk score model demonstrates strong prognostic ability. (a) Cox regression was performed to analyze the impact of age, T stage, N stage, and risk score in The Cancer Genome Atlas (TCGA)‐triple‐negative breast cancer (TNBC) cohort. (b) The precision of prognostic prediction was evaluated using calibration curves. (c) The nomogram was subjected to decision curve analysis. (d) A risk‐score‐based nomogram for TNBC patients.

The “Survminer” R package determined the optimal cut‐off value and assigned all patients to the high‐ or low‐risk groups. Patients classified as high‐risk had a considerably shorter OS compared with the low‐risk patients in both TCGA‐TNBC training and validation data sets (Figure 6c). Moreover, an assessment of the risk level for every individual was conducted by evaluating the expression of six genes through the application of regression coefficients in a Cox model (Figure 7c–d).

**Figure 8:**
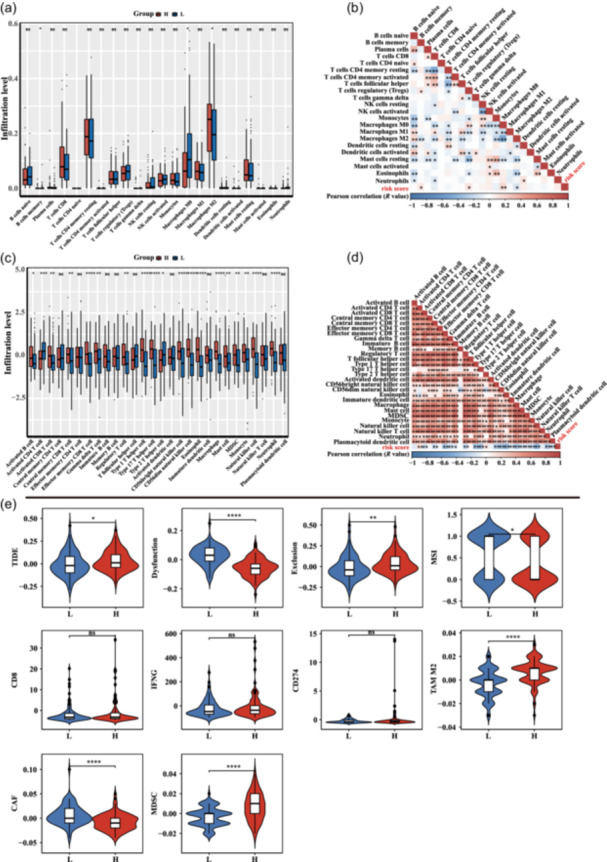
Differential immune response between high‐ and low‐risk groups. (a, c) Variation in the proportion of immune cells between high‐ and low‐risk groups. (b, d) The association between risk score and immune cell fraction. (e) Variation in immune prediction response between the two groups. *p < 0.05, **p < 0.01, ***p < 0.001, ****p < 0.0001.

To differentiate the high‐ and low‐risk groups on the basis of immune cell infiltration, we conducted differential and correlation analyses of the immune cell and immune score parameters (Figure 8a–d). We found notable variations in immune infiltration score between the two risk categories. For instance, the high‐risk group exhibited a considerably greater abundance of M2 macrophages than the low‐risk group (Figure 8a–d), whereas the low‐risk group exhibited a higher abundance of activated B cells than the high‐risk group (Figure 8c). The TIDE algorithm was used to show that myeloid‐derived suppressor cell (MDSC) numbers and TIDE scores were elevated in the high‐risk group versus low‐risk group (Figure 8d). This may seem contradictory and reflects the multifaceted nature of immune regulation.

**Figure 9:**
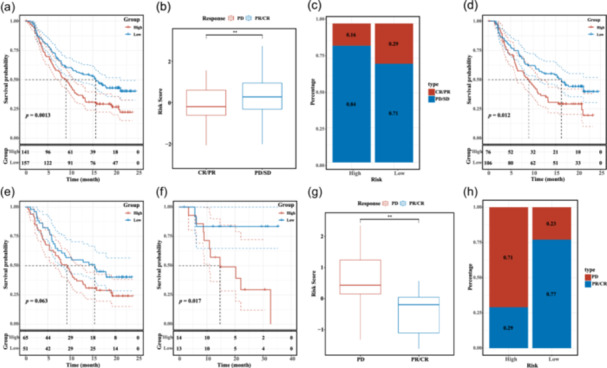
Immunotherapy response between high‐ and low‐risk groups based on GSE78220, IMvigor210. (a, d–f) Relationship between survival and risk score or prognosis. (b, c, g, h) Correlation between risk score and response to immunotherapy. **p < 0.01.

To explain the conflicting phenomenon described above, we next interrogated the immunotherapy data sets from the KEYNOTE‐052 (pembrolizumab), IMvigor210 (atezolizumab), and CheckMate 275 (nivolumab) trials. Our results confirmed the possible connection between molecular subtypes and the response to immune checkpoint inhibitors. Moreover, we found that individuals belonging to the low‐risk category exhibited a greater median survival duration and a more favorable outlook compared with those in the high‐risk score category (Figure 9a,d–f). In addition, the low‐risk score group had a higher proportion of patients with a partial response or complete response, while the high‐risk score group had a higher proportion of patients with stable disease of progressive disease (Figure 9b,c,g,h). These results validated the prognostic value of LRGs in the clinical immunotherapy data sets.

### 3.5 Lactate affects the sensitivity of multiple drugs

**Figure 10:**
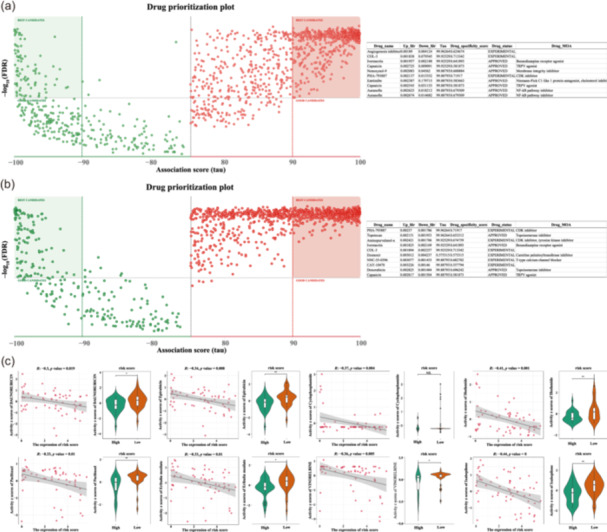
Association between lactate and various therapeutic agents. (a, b) Relationship between lactate and various therapeutic agent based on the expression profile similarity score; drug data were derived from the DREIMT database. (c) Analysis of the relationship between different groups (high‐ or low‐risk group) and drugs commonly used the clinical. *p < 0.05, **p < 0.01.

We next used DREIMT to determine the interactions between lactate and multiple pharmaceutical drugs. The results showed that the inhibitors of (cyclin‐dependent kinase) CDK, tyrosine kinase, topoisomerase, and the nuclear factor‐κB (NF‐κB) pathway interacted significantly with lactate. To further explore the association between LRGs and the drug sensitivity of TNBC, we obtained sensitivity data for TNBC neoadjuvant or adjuvant therapy drugs commonly used in clinical treatment from the CellMiner database [38]. We found that the LRGs negatively correlated with the sensitivity of TNBC to drugs such as anthracyclines, taxanes, and microtubule inhibitors, which are commonly used in clinical practice; moreover, significant statistical differences were observed between the high‐ and low‐risk groups (Figure 10c). These findings could potentially serve as a valuable resource for the development of personalized treatment plans.

## 4 DISCUSSION

TNBC is a global public health problem, and early diagnosis and treatment are crucial for improving patient prognosis [39]. Our study suggested that targeting LRGs may have potential advantages in the treatment of TNBC.

The lactate generated through the aerobic glycolysis of tumors has a wide‐ranging effect on the energy metabolism of tumors and the function and composition of immune cells in the TME [40, 41]. Unlike melanoma and lung cancer, TNBC is not overly responsive to immunotherapy and is typically still treated using chemotherapy [42]. This can be explained by the fact that TNBC has a higher tumor mutational burden and a more immunosuppressive TME than these other BC subtypes [43]. Encouragingly, we found that genes associated with IL‐17 signaling were significant enriched in the MDA‐MB‐231 cells treated with 2‐DG. Given that IL‐17 is predominantly expressed by Th17 cells, we next focused on how lactate levels altered Th17 cell dynamics in TNBC.

We therefore classified TNBC cells based on their LRG signatures and found that TNBC could be divided into two subtypes on this basis. Interestingly, we found that the two TNBC subtypes had different immune tumor environments. Subtype B was characterized by high numbers of cancer‐suppressing immune cells (e.g., activated CD8^+^ and CD4^+^ T cells), whereas Subtype A was enriched in cancer‐promoting immune cells (e.g., MDSCs and regulatory T cells [Tregs]). We next created a risk scoring system for TNBC based on the two LRG signatures. MDSCs and Tregs were enriched in the high‐risk group of TNBC patients, whereas activated CD8^+^ and CD4^+^ T cells were enriched in the low‐risk group. The fact that the LRG signatures could be used to predict the prognosis of patients with TNBC and provide an indication of their TME suggested that it could also potentially be used to predict their response to immunotherapy.

We next used the DREIMT database to obtain a list of lactate‐related therapeutic targets in TNBC based on the principle of expression profile similarity. The results suggested that certain therapeutic agents, such as CDK, tyrosine kinase, topoisomerase, and NF‐κB pathway inhibitors could potentially be used to treat TNBC patients with high lactate levels.

This study has several limitations. As this study relied solely on the analysis of bioinformatics data sets, our findings will need to be validated using functional experiments. Moreover, given that our analysis was retrospective and based on public databases, future prospective studies should be conducted to explore the mechanisms underlying lactate metabolism in TNBC and evaluate the predictive effectiveness of our risk score model.

## 5 CONCLUSIONS

We discovered 14 LRGs, which could be used to categorize patients with TNBC into high‐risk and low‐risk groups, with unique immune TME characteristics. Additionally, this LRG signature was used to develop a prognostic index, which could potentially be used to predict the response of a patient with TNBC to immunotherapy. Collectively, our findings may help implement personalized treatments for patients with TNBC.

## AUTHOR CONTRIBUTIONS

**Kaixiang Feng** and **Youcheng Shao**: Conceptualization (lead); methodology (equal); formal analysis (equal); visualization (equal); writing (lead). **Jun Li**: Data curation (equal). **Xiaoqing Guan**: Conceptualization (equal); methodology (equal). **Qin Liu**: Methodology (equal). **Meishun Hu**: Software (equal); visualization (equal). **Mengfei Chu**: Data curation (equal). **Hui Li**: Data curation (equal). **Fangfang Chen**: Supervision (equal); validation (equal). **Zongbi Yi**: Conceptualization (equal); funding acquisition (lead); supervision (equal); validation (equal). **Jingwei Zhang**: Funding acquisition (lead); supervision (equal); validation (equal).

## CONFLICT OF INTEREST STATEMENT

The authors declare no conflict of interest.

## ETHICS STATEMENT

Not applicable.

## INFORMED CONSENT

Not applicable.

## Supporting information

Supporting Information

## Data Availability

The raw data sets used in this study can be obtained from TCGA, METABRIC, and GEO. Raw RNA‐seq data were uploaded onto GEO (ID: GSE246022).

## References

[1] Siegel RL , Giaquinto AN , Jemal A . Cancer statistics, 2024. CA Cancer J Clin. 2024;74(1):12–49. 10.3322/caac.21820 38230766

[2] Bianchini G , Balko JM , Mayer IA , Sanders ME , Gianni L . Triple‐negative breast cancer: challenges and opportunities of a heterogeneous disease. Nat Rev Clin Oncol. 2016;13(11):674–690. 10.1038/nrclinonc.2016.66 27184417 PMC5461122

[3] Leon‐Ferre RA , Goetz MP . Advances in systemic therapies for triple negative breast cancer. BMJ. 2023;381:e071674. 10.1136/bmj-2022-071674 37253507

[4] Bianchini G , De Angelis C , Licata L , Gianni L . Treatment landscape of triple‐negative breast cancer‐expanded options, evolving needs. Nat Rev Clin Oncol. 2022;19(2):91–113. 10.1038/s41571-021-00565-2 34754128

[5] Jézéquel P , Loussouarn D , Guérin‐Charbonnel C , Campion L , Vanier A , Gouraud W , et al. Gene‐expression molecular subtyping of triple‐negative breast cancer tumours: importance of immune response. Breast Cancer Res. 2015;17(1):43. 10.1186/s13058-015-0550-y 25887482 PMC4389408

[6] Rosario SR , Long MD , Affronti HC , Rowsam AM , Eng KH , Smiraglia DJ . Pan‐cancer analysis of transcriptional metabolic dysregulation using The Cancer Genome Atlas. Nat Commun. 2018;9:5330. 10.1038/s41467-018-07232-8 30552315 PMC6294258

[7] Gong Y , Ji P , Yang YS , Xie S , Yu TJ , Xiao Y , et al. Metabolic‐pathway‐based subtyping of triple‐negative breast cancer reveals potential therapeutic targets. Cell Metab. 2021;33(1):51–64.e9. 10.1016/j.cmet.2020.10.012 33181091

[8] Ghergurovich JM , Lang JD , Levin MK , Briones N , Facista SJ , Mueller C , et al. Local production of lactate, ribose phosphate, and amino acids by human triple‐negative breast cancer. Med. 2021;2(6):736–754.e6. 10.1016/j.medj.2021.03.009 34223403 PMC8248508

[9] Kanaan YM , Sampey BP , Beyene D , Esnakula AK , Naab TJ , Ricks‐Santi LJ , et al. Metabolic profile of triple‐negative breast cancer in African‐American women reveals potential biomarkers of aggressive disease. Cancer Genomics Proteomics. 2014;11(6):279–294. 25422359

[10] WARBURG O . On the origin of cancer cells. Science. 1956;123(3191):309–314. 10.1126/science.123.3191.309 13298683

[11] Li Z , Cui J . Targeting the lactic acid metabolic pathway for antitumor therapy. Mol Ther Oncolytics. 2023;31:100740. 10.1016/j.omto.2023.100740 38033399 PMC10682057

[12] Brown TP , Bhattacharjee P , Ramachandran S , Sivaprakasam S , Ristic B , Sikder MOF , et al. The lactate receptor GPR81 promotes breast cancer growth via a paracrine mechanism involving antigen‐presenting cells in the tumor microenvironment. Oncogene. 2020;39(16):3292–3304. 10.1038/s41388-020-1216-5 32071396

[13] Chen P , Zuo H , Xiong H , Kolar MJ , Chu Q , Saghatelian A , et al. Gpr132 sensing of lactate mediates tumor‐macrophage interplay to promote breast cancer metastasis. Proc Natl Acad Sci USA. 2017;114(3):580–585. 10.1073/pnas.1614035114 28049847 PMC5255630

[14] Ge W , Meng L , Cao S , Hou C , Zhu X , Huang D , et al. The SIX1/LDHA axis promotes lactate accumulation and leads to NK cell dysfunction in pancreatic cancer. J Immunol Res. 2023;2023:1–21. 10.1155/2023/6891636 PMC10022590 36937004

[15] Watson MJ , Vignali PDA , Mullett SJ , Overacre‐Delgoffe AE , Peralta RM , Grebinoski S , et al. Metabolic support of tumour‐infiltrating regulatory T cells by lactic acid. Nature. 2021;591(7851):645–651. 10.1038/s41586-020-03045-2 33589820 PMC7990682

[16] Zhang D , Tang Z , Huang H , Zhou G , Cui C , Weng Y , et al. Metabolic regulation of gene expression by histone lactylation. Nature. 2019;574(7779):575–580. 10.1038/s41586-019-1678-1 31645732 PMC6818755

[17] Gu J , Zhou J , Chen Q , Xu X , Gao J , Li X , et al. Tumor metabolite lactate promotes tumorigenesis by modulating MOESIN lactylation and enhancing TGF‐β signaling in regulatory Tcells. Cell Rep. 2022;39(12):110986. 10.1016/j.celrep.2022.110986 35732125

[18] Fan W , Wang X , Zeng S , Li N , Wang G , Li R , et al. Global lactylome reveals lactylation‐dependent mechanisms underlying T_H_17 differentiation in experimental autoimmune uveitis. Sci Adv. 2023;9(42):eadh4655. 10.1126/sciadv.adh4655 37851814 PMC10584346

[19] Colaprico A , Silva TC , Olsen C , Garofano L , Cava C , Garolini D , et al. TCGAbiolinks: an R/Bioconductor package for integrative analysis of TCGA data. Nucleic Acids Res. 2016;44(8):e71. 10.1093/nar/gkv1507 26704973 PMC4856967

[20] Gao J , Aksoy BA , Dogrusoz U , Dresdner G , Gross B , Sumer SO , et al. Integrative analysis of complex cancer genomics and clinical profiles using the cBioPortal. Sci Signal. 2013;6(269):pl1. 10.1126/scisignal.2004088 23550210 PMC4160307

[21] Barrett T , Wilhite SE , Ledoux P , Evangelista C , Kim IF , Tomashevsky M , et al. NCBI GEO: archive for functional genomics data sets−update. Nucleic Acids Res. 2013;41(D1):991–995. 10.1093/nar/gks1193 PMC3531084 23193258

[22] Liberzon A , Birger C , Thorvaldsdóttir H , Ghandi M , Mesirov JP , Tamayo P . The Molecular Signatures Database hallmark gene set collection. Cell Syst. 2015;1(6):417–425. 10.1016/j.cels.2015.12.004 26771021 PMC4707969

[23] Yu G , Wang LG , Han Y , He QY . clusterProfiler: an R package for comparing biological themes among gene clusters. OMICS J Integr Biol. 2012;16(5):284–287. 10.1089/omi.2011.0118 PMC3339379 22455463

[24] Zhou Y , Zhou B , Pache L , Chang M , Khodabakhshi AH , Tanaseichuk O , et al. Metascape provides a biologist‐oriented resource for the analysis of systems‐level datasets. Nat Commun. 2019;10(1):1523. 10.1038/s41467-019-09234-6 30944313 PMC6447622

[25] Hänzelmann S , Castelo R , Guinney J . GSVA: gene set variation analysis for microarray and RNA‐Seq data. BMC Bioinformatics. 2013;14(1):7. 10.1186/1471-2105-14-7 23323831 PMC3618321

[26] Troulé K , López‐Fernández H , García‐Martín S , Reboiro‐Jato M , Carretero‐Puche C , Martorell‐Marugán J , et al. DREIMT: a drug repositioning database and prioritization tool for immunomodulation. Bioinformatics. 2021;37(4):578–579. 10.1093/bioinformatics/btaa727 32818254

[27] Wilkerson MD , Hayes DN . ConsensusClusterPlus: a class discovery tool with confidence assessments and item tracking. Bioinformatics. 2010;26(12):1572–1573. 10.1093/bioinformatics/btq170 20427518 PMC2881355

[28] Jiang P , Gu S , Pan D , Fu J , Sahu A , Hu X , et al. Signatures of T cell dysfunction and exclusion predict cancer immunotherapy response. Nat Med. 2018;24(10):1550–1558. 10.1038/s41591-018-0136-1 30127393 PMC6487502

[29] Mariathasan S , Turley SJ , Nickles D , Castiglioni A , Yuen K , Wang Y , et al. TGFβ attenuates tumour response to PD‐L1 blockade by contributing to exclusion of T cells. Nature. 2018;554(7693):544–548. 10.1038/nature25501 29443960 PMC6028240

[30] Hugo W , Zaretsky JM , Sun L , Song C , Moreno BH , Hu‐Lieskovan S , et al. Genomic and transcriptomic features of response to anti‐PD‐1 therapy in metastatic melanoma. Cell. 2017;168(3):542. 10.1016/j.cell.2017.01.010 28129544

[31] Li T , Fan J , Wang B , Traugh N , Chen Q , Liu JS , et al. TIMER: a web server for comprehensive analysis of tumor‐infiltrating immune cells. Cancer Res. 2017;77(21):e108–e110. 10.1158/0008-5472.CAN-17-0307 29092952 PMC6042652

[32] Becht E , Giraldo NA , Lacroix L , Buttard B , Elarouci N , Petitprez F , et al. Estimating thepopulation abundance of tissue‐infiltrating immune and stromal cell populations using gene expression. Genome Biol. 2016;17(1):218. 10.1186/s13059-016-1070-5 27765066 PMC5073889

[33] Chen B , Khodadoust MS , Liu CL , Newman AM , Alizadeh AA . Profiling tumor infiltrating immune cells with CIBERSORT. Meth Mol Biol Clifton N J. 2018;1711:243–259. 10.1007/978-1-4939-7493-1_12 PMC5895181 29344893

[34] Racle J , Gfeller D . EPIC: a tool to estimate the proportions of different cell types from bulk gene expression data. Meth Mol Biol Clift N J. 2020;2120:233–248. 10.1007/978-1-0716-0327-7_17 32124324

[35] Zeng D , Ye Z , Shen R , Yu G , Wu J , Xiong Y , et al. IOBR: multi‐omics immuno‐oncology biological research to decode tumor microenvironment and signatures. Front Immunol. 2021;12:687975. 10.3389/fimmu.2021.687975 34276676 PMC8283787

[36] Langfelder P , Horvath S . WGCNA: an R package for weighted correlation network analysis. BMC Bioinformatics. 2008;9(1):559. 10.1186/1471-2105-9-559 19114008 PMC2631488

[37] Liu Z , Liu L , Weng S , Guo C , Dang Q , Xu H , et al. Machine learning‐based integration develops an immune‐derived lncRNA signature for improving outcomes in colorectal cancer. Nat Commun. 2022;13:816. 10.1038/s41467-022-28421-6 35145098 PMC8831564

[38] Shankavaram UT , Varma S , Kane D , Sunshine M , Chary KK , Reinhold WC , et al. CellMiner: a relational database and query tool for the NCI‐60 cancer cell lines. BMC Genomics. 2009;10(1):277. 10.1186/1471-2164-10-277 19549304 PMC2709662

[39] Vagia E , Mahalingam D , Cristofanilli M . The landscape of targeted therapies in TNBC. Cancers. 2020;12(4):916. 10.3390/cancers12040916 32276534 PMC7226210

[40] Faubert B , Li KY , Cai L , Hensley CT , Kim J , Zacharias LG , et al. Lactate metabolism in human lung tumors. Cell. 2017;171(2):358–371.e9. 10.1016/j.cell.2017.09.019 28985563 PMC5684706

[41] Frisardi V , Canovi S , Vaccaro S , Frazzi R . The significance of microenvironmental and circulating lactate in breast cancer. Int J Mol Sci. 2023;24(20):15369. 10.3390/ijms242015369 37895048 PMC10607673

[42] Agostinetto E , Losurdo A , Nader‐Marta G , Santoro A , Punie K , Barroso R , et al. Progress and pitfalls in the use of immunotherapy for patients with triple negative breast cancer. Expert Opin Invest Drugs. 2022;31(6):567–591. 10.1080/13543784.2022.2049232 35240902

[43] He Y , Jiang Z , Chen C , Wang X . Classification of triple‐negative breast cancers based on immunogenomic profiling. J Exp Clin Cancer Res. 2018;37(1):327. 10.1186/s13046-018-1002-1 30594216 PMC6310928

