# Supplementary material for: A lactate‐responsive gene signature predicts the prognosis and immunotherapeutic response of patients with triple‐negative breast cancer
Source: Cancer Innov. 2024 May 17;3(4):e124. doi: 10.1002/cai2.124 (PMC11212277; doi:10.1002/cai2.124)
Supplement: Supplementary file 1 — Supporting Information [file CAI2-3-e124-s001.docx]

**Supplementary Table1 lactate metabolism-related gene sets from the Molecular Signatures Database (MsigDB)**

| **Name** | **Number** | **Description** | **Collections** | **Contributor** |
| --- | --- | --- | --- | --- |
| **GOBP_LACTATE_METABOLIC_PROCESS** | 18 | The chemical reactions and pathways involving lactate, the anion of lactic acid. [ISBN:0198547684] | C5 GO | Gene Ontology Consortium |
| **GOBP_LACTATE_TRANSMEMBRANE_TRANSPORT** | 7 | The process in which lactate is transported across a membrane. Lactate is 2-hydroxypropanoate, CH3-CHOH-COOH; L(+)-lactate is formed by anaerobic glycolysis in animal tissues, and DL-lactate is found in sour milk, molasses and certain fruit juices. [GOC:mcc, ISBN:0198506732] | C5 GO | Gene Ontology Consortium |
| **GOMF_LACTATE_DEHYDROGENASE_ACTIVITY** | 6 | Catalysis of the reaction: lactate + NAD+ = H+ + NADH + pyruvate. [GOC:ai, GOC:bf] | C5 GO | Gene Ontology Consortium |
| **GOMF_LACTATE_TRANSMEMBRANE_TRANSPORTER_A CTIVITY** | 6 | Enables the transfer of lactate from one side of a membrane to the other. Lactate is 2-hydroxypropanoate, CH3-CHOH-COOH; L(+)-lactate is formed by anaerobic glycolysis in animal tissues, and DL-lactate is found in sour milk, molasses and certain fruit juices. [GOC:ai, ISBN:0198506732, RHEA:34987] | C5 GO | Gene Ontology Consortium |
| **HP_ABNORMAL_BRAIN_LACTATE_LEVEL_BY_MRS** | 39 | Abnormal brain lactate level by MRS | C5 | The Jackson Laboratory (JAX) |
| **HP_ABNORMAL_LACTATE_DEHYDROGENASE_LEVEL** | 91 | Abnormal lactate dehydrogenase level | C5 | The Jackson Laboratory (JAX) |
| **HP_ELEVATED_LACTATE_PYRUVATE_RATIO** | 21 | Elevated lactate:pyruvate ratio | C5 | The Jackson Laboratory (JAX) |
| **HP_INCREASED_CIRCULATING_LACTATE_DEHYDRO GENASE_CONCENTRATION** | 71 | Increased circulating lactate dehydrogenase concentration | C5 | The Jackson Laboratory (JAX) |
| **HP_INCREASED_CSF_LACTATE** | 113 | Increased CSF lactate | C5 | The Jackson Laboratory (JAX) |
| **HP_INCREASED_SERUM_LACTATE** | 219 | Increased serum lactate | C5 | The Jackson Laboratory (JAX) |
| **HP_LACTIC_ACIDOSIS** | 198 | Lactic acidosis | C5 | The Jackson Laboratory (JAX) |
| **HP_LACTICACIDURIA** | 30 | Lacticaciduria | C5 | The Jackson Laboratory (JAX) |
| **HP_SEVERE_LACTIC_ACIDOSIS** | 14 | Severe lactic acidosis | C5 | The Jackson Laboratory (JAX) |
| **KEGG_MEDICUS_REFERENCE_GLYCOLYSIS** | 25 | Pathway Definition from KEGG: Glc-6P -- GPI >> PFK >> ALDO >> GAPDH >> PGK1/2 >> (PGAM,BPGM) >> ENO1/2/3/4 >> (PKLR,PKM) >> (LDH,LDHAL6) -> Lactate | C2 CP | Kyoto Encyclopedia of Genes and Genomes |
| **WP_LACTATE_SHUTTLE_IN_GLIAL_CELLS** | 13 | Lactate shuttle in glial cells | C2 CP | WikiPathways |

**Note: we searched and filtered with “lactate”, “lactic acid” as the keyword to collect** **lactate metabolism-related gene sets from the Molecular Signatures Database (MsigDB). The red marker represent gene set including in this study; black represent gene set excluded in this study.**
